# Use of a Population-Based Cancer Registry to Calculate Twenty-Year Trends in Cancer Incidence and Mortality in Fukui Prefecture

**DOI:** 10.2188/jea.JE20090102

**Published:** 2010-05-05

**Authors:** Masakazu Hattori, Manabu Fujita, Yuri Ito, Akiko Ioka, Kota Katanoda, Yosikazu Nakamura

**Affiliations:** 1 Department of Cancer Therapy Center, Fukui Prefectural Hospital, Fukui, Japan; 2 Department of Internal Medicine, Fukui Social Insurance Hospital, Katsuyama, Fukui, Japan; 3 Department of Cancer Control and Statistics, Osaka Medical Center for Cancer and Cardiovascular Diseases, Osaka, Japan; 4 Cancer Information Services and Surveillance Division, Center for Cancer Control and Information Services, National Cancer Center, Tokyo, Japan; 5 Department of Public Health, Jichi Medical University, Shimotsuke, Japan

**Keywords:** incidence, mortality, population-based cancer registry

## Abstract

**Background:**

There have been only a limited number of trend analyses of incidence and mortality using population-based cancer registry data in Japan, and the national statistics regarding incidence are estimated data. In the present study, data from the Fukui Prefecture cancer registry, which is the most accurate in Japan, were used to observe trends in incidence and mortality rates.

**Methods:**

Cancer incidence and mortality rates from 1984 through 2004 were obtained from the Fukui Prefecture cancer registry. Joinpoint analysis developed for the US National Cancer Institute’s Surveillance, Epidemiology, and End Results (SEER) Program was used to compute and graphically present annual percentage changes in age-adjusted incidence and mortality in Fukui Prefecture.

**Results:**

On joinpoint analysis, there were slight increases in incidence at all cancer sites combined for both sexes from 1986, but the trend was not significant in Fukui. Mortality in women appeared to significantly decrease, while mortality in men, which had been increasing until 1999, began to significantly decrease thereafter. In an analysis by anatomical site, both the incidence and mortality of stomach cancer significantly decreased in both sexes. However, the incidence and mortality of breast and prostate cancers significantly increased. The mortality of liver and lung cancers also increased in both sexes.

**Conclusions:**

Cancer mortality has been declining in recent years, and the reduction in mortality from stomach cancer has significantly affected the trends in Fukui. Urgent cancer control planning by the Fukui local government is necessary, especially for cancers of the liver, lung, prostate, and breast.

## INTRODUCTION

In Japan, cancer has been the leading cause of death since 1981.^1^ To establish a comprehensive system to combat cancer, the “Fundamental Law for Anti-Cancer Measures” was enacted in June 2006, and, in March 2008, a “Plan to Promote Anti-Cancer Programs in Fukui Prefecture^2^” was formulated. Fukui Prefecture aimed to be a model district in the prevention and treatment of cancer, and also set a goal of reducing age-adjusted cancer mortality by 20% within the next 10 years for residents younger than 75 years. To obtain accurate data on cancer incidence and mortality—and for precise evaluation of cancer control—a highly accurate population-based cancer registry was required. Among the cancer registries already established in 34 prefectures and in municipal districts in Japan, only 6^3^ (including the one in Fukui Prefecture) have achieved an international level of reliability, which is defined as a death certificate only (DCO) percentage <25% and an incidence/death ratio ≥1.5.^4^^,^^5^ The trends observed in the Japanese nationwide incidence are based on estimates from these registries, so those data affect the level of reliability. The Fukui Prefecture Cancer Registry, which was started in 1984 under the auspices of the Fukui Medical Association, has had a DCO percentage of approximately 5%^6^ every year since 1987, and boasts a level of accuracy comparable to that of the United States,^4^ where statistics are compiled by law. In the current study, instead of trend analysis based on estimated data, changes in age-adjusted incidence and mortality rates derived from the highly accurate population-based cancer registry data were investigated and the results were analyzed.

In Fukui Prefecture, which has a short history of registration and a low population (820 000) that has been stable for 25 years, it was difficult to obtain sequential trends from cancer statistics, which require many years of data, and compare the results with national statistics. At present, ie, 25 years after the inception of the registration system, it is now possible to observe annual trends in incidence and mortality. We aimed to clarify the differences in cancer trends between Fukui Prefecture and national statistics and to analyze the reasons for these differences.

## METHODS

Incidence and mortality statistics were obtained from the Fukui Prefecture Cancer Registry data^6^ for the period from 1984 through 2004 (mortality data for 2005 and 2006 were not available because of the development of a new database system in Fukui Prefecture). Age-adjusted incidence and mortality rates (ASRs, per 100 000) were computed from these statistics. The Japanese model population in 1985 was used as the standard population. For national data, the incidence rate was computed from the estimated national data, which was assembled by the Research Group for Population-Based Cancer Registration in Japan, Ministry of Health, Labour and Welfare, and compiled by the National Cancer Center^7^ for the years from 1975 through 2002. The national mortality rate was computed based on published data from the National Vital Statistics of Japan from 1958 through 2006.^1^

To analyze annual trends from Fukui and the national data, joinpoint analysis was used for computation and graphical representation. The software was developed by the Surveillance, Epidemiology and End Results Program (SEER) of the US National Cancer Institute (NCI)^8^ to capture sequential changes in statistics such as incidence and mortality. This method describes changes in data trends by connecting several different line segments on a log scale at joinpoints. Analysis starts with 0 joinpoints (representing a straight line) and tests for model fit with a maximum of 3 joinpoints. The Monte Carlo Permutation method in the same program^8^ was used to analyze the significance of any differences. Annual percentage change (APC) in age-adjusted incidence, mortality rate for each line segment, and the corresponding 95% confidence interval were estimated. The APC was tested with constant variance to determine whether there was a difference from no change (0%). Each joinpoint informs a statistically significant change in trend (increase or decrease) and each of those trends are described by the APC.^8^^,^^9^

The cancer sites to be evaluated were all sites combined, as indicated by the International Classification of Diseases, 10th Revised Version (ICD-10), and individual sites, ie, the stomach (C16), colon and rectum (C18–C21), liver (C22), lung (C33–C34), breast (C50), uterus (C53–C55), and prostate (C61).

## RESULTS

**Figure 1.**
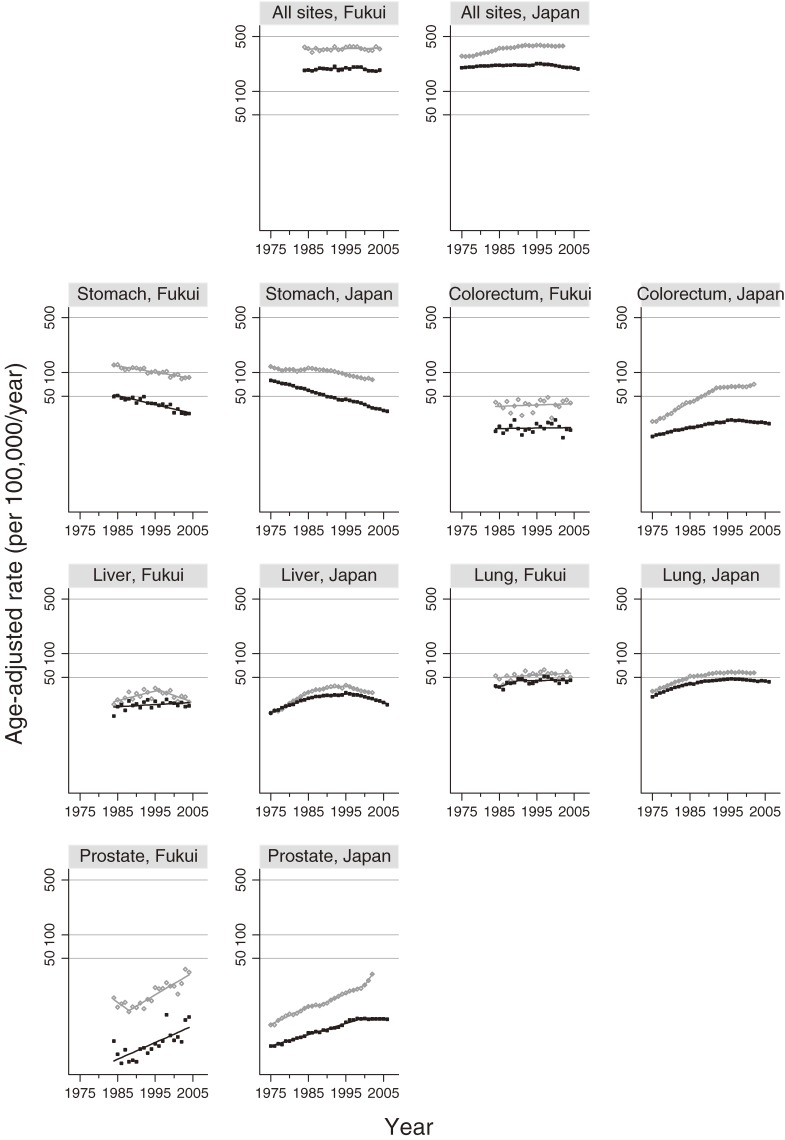
Trends for age-adjusted incidence and mortality (per 100 000) among men for all cancer sites combined (ICD-10 C00–C96), stomach cancer (C16), colorectal cancer (excluding carcinoma in situ, C18–C21), liver cancer (C22), lung cancer (C33–C34), and prostate cancer (C61). Age was adjusted by using direct methods with the Japanese model population in 1985. The gray line represents incidence and the black line represents mortality. ^a^APC is significantly different from 0 (*P* < 0.05).

**Figure 2.**
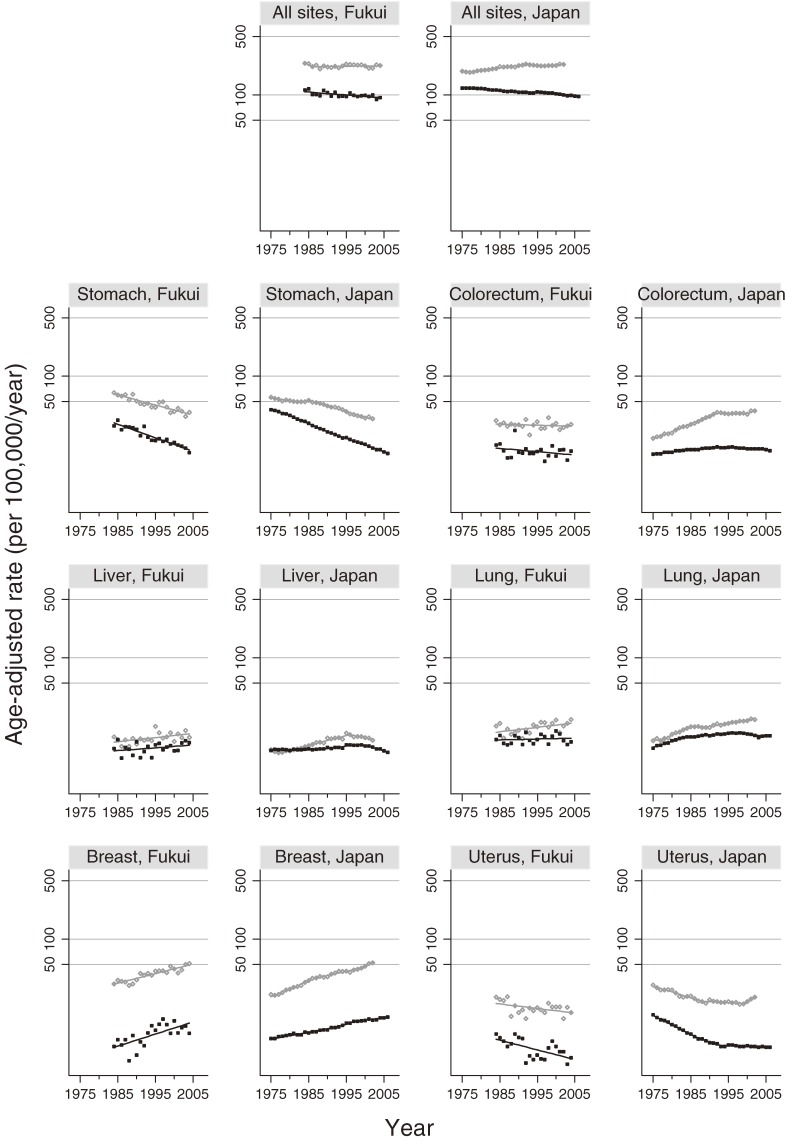
Trends for age-adjusted incidence and mortality (per 100 000) among women for all cancer sites combined (ICD-10 C00–C96), stomach cancer (C16), colorectal cancer (excluding carcinoma in situ, C18–C21), liver cancer (C22), lung cancer (C33–C34), breast cancer (C50), and uterine cancer (C53–C55). Age was adjusted by using direct methods with the Japanese model population in 1985. The gray line represents incidence and the black line represents mortality. ^a^APC is significantly different from 0 (*P* < 0.05).

Tables 1 and 2 show the APCs and trends in incidence and mortality rates at all sites combined (ICD-10, C00–C96, divided by sex) and at individual sites in Fukui Prefecture and in national estimates. Figures 1 and 2 show the trends in incidence and mortality rates at all sites combined and at individual sites in Fukui Prefecture and in national estimates. For cancer at all sites combined among men, the incidences in Fukui Prefecture formed a plateau (not significant), while for mortality in Fukui Prefecture there was a significant decreasing trend starting in 1999. Regarding incidence in national data, there was a significant increasing trend up to 1992, but from 1992 through 2002, the rate significantly decreased; mortality in national data showed a significant increase up to 1997, but in the period from 1997 through 2006 the APC changed significantly to −1.55. For cancer at all sites combined among women in Fukui, incidence increased somewhat (APC, 0.12; not significant) in the data and figures, but mortality significantly decreased from 1984. In national data, the APC for incidence in 1997 through 2002 was 0.93 (not significant), and the APC (−1.20) for mortality in 1996–2006 indicated that there was a significant decrease.

**Table 1.** Trends in cancer incidence and mortality in Fukui, as determined by joinpoint regression analysis (men)

**Incidence**		Line Segment 1		Line Segment 2		Line Segment 3		Line Segment 4	
					
site		Years	APC	Years	APC	Years	APC	Years	APC
All sites	Fukui	1984–2004	0.14						
	*Japan*	1975–1978	0.44	1978–1985	3.00^a^	1985–1992	1.46^a^	1992–2002	−0.20^a^
Stomach	Fukui	1984–2004	−1.81^a^						
	*Japan*	1975–1978	−3.50^a^	1978–1988	0.37	1988–2002	−2.27^a^		
Colorectum	Fukui	1984–2004	0.39						
	*Japan*	1975–1992	6.13^a^	1992–2002	0.91^a^				
Liver	Fukui	1984–1996	2.87^a^	1996–2004	−3.90^a^				
	*Japan*	1975–1986	6.60^a^	1986–1995	1.46^a^	1995–2002	−3.18^a^		
Lung	Fukui	1984–2004	0.67						
	*Japan*	1975–1985	4.47^a^	1985–1995	1.38^a^	1995–2002	−0.32		
Prostate	Fukui	1984–1988	−7.59	1988–2004	6.77^a^				
	*Japan*	1975–1985	5.46^a^	1985–1988	1.17	1988–2000	5.03^a^	2000–2002	17.02^a^

**Mortality**		Line Segment 1		Line Segment 2		Line Segment 3		Line Segment 4	
					
site		Years	APC	Years	APC	Years	APC	Years	APC

All sites	Fukui	1984–1999	0.54^a^	1999–2004	−1.84^a^				
	*Japan*	1958–1964	1.05^a^	1964–1997	0.41^a^	1997–2006	−1.55^a^		
Stomach	Fukui	1984–2004	−2.60^a^						
	*Japan*	1958–1971	−0.69^a^	1971–1993	−3.05^a^	1993–1996	−0.99	1996–2006	−3.37^a^
Colorectum	Fukui	1984–2004	0.16						
	*Japan*	1958–1960	−0.07	1960–1980	3.16^a^	1980–1996	2.03^a^	1996–2006	−0.96^a^
Liver	Fukui	1984–2004	0.66						
	*Japan*	1958–1974	−0.63^a^	1974–1986	4.31^a^	1986–1997	0.86^a^	1997–2006	−3.26^a^
Lung	Fukui	1984–1990	3.69^a^	1990–2004	0.04				
	*Japan*	1958–1963	7.72^a^	1963–1983	4.51^a^	1983–1996	1.37^a^	1996–2006	−0.95^a^
Prostate	Fukui	1984–2004	5.04^a^						
	*Japan*	1958–1999	3.41^a^	1999–2006	−0.09				

Annualized period—incidence: 1984–2004, mortality: 1984–2004.

^a^Annual percentage change (APC) significantly different from 0 (*P* < 0.05).

**Table 2.** Trends in cancer incidence and mortality in Fukui, as determined by joinpoint regression analysis (women)

**Incidence**		Line Segment 1		Line Segment 2		Line Segment 3		Line Segment 4	
					
site		Years	APC	Years	APC	Years	APC	Years	APC
All sites	Fukui	1984–1986	−5.13	1986–2004	0.12				
	*Japan*	1975–1977	−0.90	1977–1992	1.41^a^	1992–1997	−0.91	1997–2002	0.93
Stomach	Fukui	1984–2004	−2.77^a^						
	*Japan*	1975–1982	−1.69^a^	1982–1985	1.67	1985–2002	−3.04^a^		
Colorectum	Fukui	1984–2004	−0.31						
	*Japan*	1975–1992	4.30^a^	1992–2000	−0.04	2000–2002	4.28		
Liver	Fukui	1984–2004	1.12^a^						
	*Japan*	1975–1977	−4.54	1977–1996	2.76^a^	1996–2002	−2.29^a^		
Lung	Fukui	1984–2004	1.23^a^						
	*Japan*	1975–1977	−0.52	1977–1985	4.82^a^	1985–1989	−0.44	1989–2002	1.77^a^
Breast	Fukui	1984–2004	2.66^a^						
	*Japan*	1975–1987	4.42^a^	1987–1999	2.25^a^	1999–2002	5.45^a^		
Uterus	Fukui	1984–2004	−1.23^a^						
	*Japan*	1975–1988	−3.16^a^	1988–1999	−0.52	1999–2002	5.97^a^		

**Mortality**		Line Segment 1		Line Segment 2		Line Segment 3		Line Segment 4	
					
site		Years	APC	Years	APC	Years	APC	Years	APC

All sites	Fukui	1984–2004	−0.81^a^						
	*Japan*	1958–1968	−0.14	1968–1993	−0.83^a^	1993–1996	0.85	1996–2006	−1.20^a^
Stomach	Fukui	1984–2004	−3.64^a^						
	*Japan*	1958–1970	−0.76^a^	1970–1979	−3.19^a^	1979–1990	−4.36^a^	1990–2006	−3.59^a^
Colorectum	Fukui	1984–2004	−0.89						
	*Japan*	1958–1963	1.29^a^	1963–1973	2.65^a^	1973–1992	1.15^a^	1992–2006	−0.57^a^
Liver	Fukui	1984–2004	0.77						
	*Japan*	1958–1975	−2.36^a^	1975–1988	0.14	1988–1999	1.26^a^	1999–2006	−2.89^a^
Lung	Fukui	1984–2004	0.22						
	*Japan*	1958–1964	5.97^a^	1964–1984	3.06^a^	1984–1997	0.91^a^	1997–2006	−1.18^a^
Breast	Fukui	1984–2004	3.37^a^						
	*Japan*	1958–1962	−1.45	1962–1993	1.93^a^	1993–1997	3.58^a^	1997–2006	1.26^a^
Uterus	Fukui	1984–2004	−2.62^a^						
	*Japan*	1958–1972	−3.23^a^	1972–1987	−5.07^a^	1987–1993	−3.39^a^	1993–2006	−0.30^a^

Annualized period—incidence: 1984–2004, mortality: 1984–2004.

^a^Annual percentage change (APC) significantly different from 0 (*P* < 0.05).

Regarding the trends for stomach cancer (C16) in Fukui Prefecture, there was a significant decrease in both incidence and mortality for men and women since 1984. In national data, the incidence in men decreased significantly since 1988; the APC (−2.27) and incidence in women significantly decreased since 1985 (APC, −3.04). Mortality in men (excluding 1993–1996; −0.99, not significant) and women significantly decreased since 1958. It should be noted that the reduction in mortality exceeded that of incidence in both the Fukui Prefecture data and national estimates, and that the APCs for incidence and mortality in both sexes were lower in Fukui than in the national statistics.

In Fukui Prefecture, colorectal cancer incidence and mortality (C18–C21) increased among men (not significant); among women, however, both incidence and mortality declined (not significant). The age-adjusted incidence rates for men and women in Fukui Prefecture (in 2002, men: 42.9; women: 24.4) were lower than those in the national statistics (in 2002, men: 70.7; women: 38.6). However, in the national estimates, there was a significant increasing trend in the incidence of colorectal cancer among both men and women up to 1992; from 1992 through 2002, the rate of increase became more gradual. Among women, however, the increasing trend reemerged from 2000 (APC, 4.28; not significant). Mortality gradually but significantly decreased from 1996 in men and 1992 in women.

In Fukui Prefecture, the incidence of liver cancer (C22, Figures 1 and 2) in men began to significantly decline in 1996, but mortality continued to rise. Among women, incidence significantly increased and mortality remain unchanged; these findings differ from the national estimates, especially regarding the mortality trend. According to the national estimates, the incidence of liver cancer changed significantly from an increase to a decrease in both men and women starting in 1995 for men and in 1996 for women. Mortality significantly declined in men since 1997 and in women since 1999.

Regarding trends for lung cancer (C33–C34), the Fukui data and figures show no significant increase in incidence and mortality among men; however, there was a significant increase in incidence among women in Fukui, although the mortality rate remained almost unchanged (not significant). In the national data and figures for lung cancer, there was a flat or slightly decreasing trend in incidence among men starting in approximately 1995 (not significant). In contrast, there was a significant increasing trend in the data and figure for women (1989–2002; APC, 1.77). Mortality, on the other hand, significantly declined among men starting from 1996 and among women starting from 1997.

There was a significantly increasing trend in incidence, and an associated increase in mortality, for breast cancer (C50) in data from Fukui and in national statistics (Table 2 and Figure 2). In Fukui Prefecture, the annual percentage change (1984–2004 APC, 3.37) in mortality was higher than that in the national data (1997–2006 APC, 1.26; Table 2).

In Fukui Prefecture, both the incidence and mortality of uterine cancer (C53–C55, Table 2 and Figure 2) significantly declined. According to the national data and the figure, the incidence of uterine cancer remained unchanged until 1999, when a significant upturn began (1999–2002 APC, 5.97). There was a difference in the incidence trends for uterine cancer, and mortality declined more rapidly in Fukui Prefecture than in national data.

In Fukui Prefecture, both the incidence and mortality of prostate cancer (C61, Table 1 and Figure 1) increased significantly. The incidence of prostate cancer, which had been on the rise in the national statistics, increased further in the 2000s (2000–2002 APC, 17.02). Mortality, on the other hand, showed a tendency to increase but leveled off starting in 1999, after which it began to slightly decrease (1999–2006 APC, −0.09). There were discrepancies between the Fukui Prefecture and national data in both incidence and mortality.

## DISCUSSION

In our calculations using joinpoint analysis, we had difficulty in analyzing the data from Fukui Prefecture during the observation period. This may have been due to the small population of this prefecture, the resultant marked data spread, and/or the short survey period; however, we remain convinced of the importance of using population-based cancer registry data, rather than estimated data, to observe annual trends in incidence and mortality.

Regarding trends for all cancer sites combined, the incidence rate increased slightly in both men and women in Fukui Prefecture, although mortality has recently declined in both sexes. In the national data, 1992 was the peak year for cancer incidence in men, among whom mortality began to decline in 1997, which indicates that there is an approximately 5-year time lag between the 2 sets of statistics. The incidence for women for all cancer sites combined began to decrease in 1992, but started to rise again in 1997. The increased incidences of colorectal (2000–), lung (1989–), uterine (1999–), and breast (1975–) cancers have been cited to explain this increase. Mortality in women showed the expected reduction, which we believe is primarily attributable to the significant reduction in the mortality of stomach cancer. We also believe that the effects of mass screening and improved diagnosis and therapeutic techniques have played a role in these findings.

Both incidence and mortality in stomach cancer significantly declined, but we noted a dissociation phenomenon, ie, an APC decrease that was greater for mortality than for incidence, in Fukui and the national estimates. In Fukui Prefecture, the ratio of early cancers (ie, cancers localized in the mucosal layer or invading the submucosal layer: early cancers/all stomach cancers) reached 47.7% in 2002 from 32.4% in 1985, and 5-year relative survival rate increased from 49.4% in 1985 to 55.9% in 2002. With respect to early diagnosis, 75% of stomach cancers detected by screening (excluding those with unknown depth of invasion) were early cancers, and the ratio of early cancers diagnosed on the basis of clinical symptoms (excluding those with unknown depth of invasion) was approximately 50% in Fukui Prefecture. Mass screening is an effective tool for early diagnosis of stomach cancer,^10^ but the percentage of eligible residents undergoing annual population-based stomach cancer mass screening by X-ray fluoroscopy has remained at approximately 15% during a recent 20-year period. With respect to the methods for detecting early stomach cancer at Fukui Prefectural Hospital, the ratio of patients referred as a result of opportunistic screening (at specific institutions) by endoscopy examination reached 56% in the period from 2000 through 2008, which was a substantial increase from the rate of 25% observed from 1982 through 1990.^11^ Therefore, we believe that the rising trend in the early cancer ratio is due to a change in the methods used for detecting early stomach cancer. Early diagnosis and early therapy play an important role in the previously mentioned dissociation phenomenon between incidence and mortality. A review of these data suggests the need for further promotion of stomach cancer screening (both population-based and opportunistic) for early diagnosis.

Regarding colorectal cancer in Fukui, the age-adjusted incidence for both sexes was lower than that in the national estimates. The Fukui registry collects data on the depth of invasion of gastrointestinal tract cancer; therefore, carcinoma in situ is excluded because it is not an invasive cancer. However, national estimates may include such neoplasms. We calculated the APC for the incidence of colorectal cancer including carcinoma in situ (APC′) in Fukui. Among men, the APC′ was 1.43 for the 10-year period beginning in 1984. This value was significantly higher than the APC of 0.39 for gastrointestinal tract cancers excluding carcinoma in situ. Among women, the APC′ for 1984–2004 was 0.51 (not significant), which was higher than the APC of −0.31. These trends were similar to the national data, which include carcinoma in situ. In Fukui Prefecture, population-based mass screening with the fecal occult blood test was started in 1987, and the percentage of eligible residents undergoing such screening was 15% in 1989 and 22% in 2004. The ratio of clinically localized cancers (cancers confined to the original organ) in colon cancer rose to 48.8% in 2002 from 32.3% in 1985; in rectal cancer the ratio increased to 46.9% in 2002 from 41.6% in 1985. The 5-year relative survival rate for people with colon cancer rose from 51.5% to 65.9% between 1985 and 2002, and that for rectal cancer rose from 56.0% to 63.1%. Early diagnosis and early treatment improved 5-year survival rates in a recent 20-year period, but the dissociation between increased incidence and decreased mortality, such as that seen in the national statistics, was not noted among either men or women. One reason why there has been no obvious difference between the incidence and mortality rates for colorectal cancer is that the annual rate of distant cancer (cancer that has metastasized to distant organs) has remained almost unchanged. In practice, the rates of distant cancer in 1985 and 2002 were 16.1% and 16.4%, respectively, for colon cancer and 14.4% and 11.8% for rectal cancer. We have not been able to reduce the number of advanced cases in the last 15 years. Therefore, we must continue to observe further trends in incidence and mortality for colorectal cancer, and to improve public participation in mass screening examinations. A new approach to screening is also necessary.

According to the national statistics, both the incidence and mortality of liver cancer have declined since the late 1990s, while in Fukui Prefecture, only incidence in men decreased. Because of the time lag, one might expect mortality among men to decrease as well. Among women, however, both incidence and mortality are increasing. In Fukui Prefecture, the DCO percentage for liver cancer has been at a plateau of about 5% and the incidence/death ratio has been about 1.1 in a recent 20-year period. Data on the incidence of liver cancer are affected by the accuracy of the cancer registry,^12^ but we believe that the Fukui liver cancer data were not affected by the reliability of registration. In Fukui, the male/female ratio was almost 3:1, and the age distribution and incidence of liver cancer were highest among people in their 70s, as they were in other Japanese prefectures.^12^ The 5-year relative survival rate increased to 21.9% in 1998 from 4.8% in 1985. Because advances in hepatitis research allow for better treatment and follow-up, survival is likely to improve. Regarding liver cancer staging, in 1994–1998 the ratio of localized cancer to distant cancer was 7.4 in Fukui, 8.9 in Yamagata, and 4.5 in Osaka (Yamagata and Osaka are included in the national data estimates).^13^ Approximately 90% of liver cancers are hepatocellular carcinomas (HCCs), which, in Japan, are mainly caused by chronic hepatitis C virus (HCV) infection rather than by chronic hepatitis B virus infection.^14^ The rate of HCV infection on screening was 1.0% in Fukui Prefecture in 2003; the overall rate in the national data was also 1.0%.^15^ We are unable to explain why liver cancer mortality has decreased in both sexes in the national data, but has not in either sex in Fukui. We must investigate whether there is a positive association between the incidence reduction and mortality reduction in men, as the incidence decreased in men. However, incidence in women continued to increase over a 20-year period, which may explain why a decline in mortality has not yet occurred. The reason for the high incidence of liver cancer in women cannot be identified in this study. Nonetheless, there is clearly a need to promote policies to combat liver cancer.

The national statistics for lung cancer incidence and mortality show a change from a plateau to a gradual reduction, except for incidence among women. However, in Fukui Prefecture, both incidence and mortality continued to rise. In particular, mortality in both sexes in Fukui differed from that reported in the national estimates. The percentage of individuals undergoing mass screening was between 27% and 30% in a recent 15-year period. The percentage of localized cancer was little changed between 1985 (16.0%) and 2002 (19.7%). The ratio of localized cancer to distant cancer in 1994–1998 was 0.77 in Fukui, 0.89 in Yamagata, and 0.52 in Osaka.^13^ In Fukui, there were no differences with respect to tendencies in the number of distant cancer cases or localized cancer cases, as compared with other prefectures, and the increase in the proportion of localized cancers was not particularly sequential. The 5-year relative survival rate in 1985 was 7.8% and improved to 18.4% in 1998. Mass screening using plain radiography is believed to be inadequate for early detection of lung cancer, and the rising survival rate is instead attributable to advances in the treatment of lung cancer. In any case, we call for the prompt development of a screening system for detecting cancer at an early stage. In 2004, the smoking rate among men was 45.8% in Fukui and 43.3% in Japan; among women, it was 7.7% in Fukui and 12.0% in Japan.^2^ Preventive measures, including antismoking campaigns, are urgently needed in Fukui Prefecture.

The incidence of breast cancer significantly increased in Fukui and in national estimates; however, the APC for the national data was higher than that for Fukui. Still, the percentage increase in breast cancer mortality was notable in Fukui Prefecture. In Fukui Prefecture, the annual percentage of eligible individuals undergoing population-based mass screening remained approximately 15% from 1989 through 2004, and the ratio of localized cancer remained almost the same from 1985 (47.4%) to 2002 (47.8%). We believe that mass screening for cancer by inspection and palpation of the breast is inadequate for early detection of this cancer. In Fukui Prefecture, mass screening using mammography started in 2003, which was too late to be reflected in our data. The 5-year relative survival rate for breast cancer between 1985 and 1998 rose from 77.6% to 84.4%. This improved survival rate resulted from recent advances in breast cancer treatment. However, mortality in Fukui was higher than in the national statistics, for several reasons. From 1994 through 1998, the 5-year survival rate for regional cancer (cancer that has spread to regional lymph nodes or immediately adjacent tissues) was 75.2%, which was lower than the rate of 80.1% for Yamagata during the same period.^13^ The localized cancer/distant cancer ratio in 1994–1998 was 8.77, which was lower than in Osaka (9.12) and Yamagata (9.61).^13^ Also, the ratio of advanced cancer in Fukui was higher than in other prefectures. These findings also highlight the need for screening policies using mammography for early diagnosis. In Fukui, mammography screening has become an accepted part of breast cancer examination, and 89.7% of examinees underwent mammography in 2007; the expectations for early detection of breast cancer are high. Further observation of temporal trends in breast cancer mortality is needed to assess the effectiveness of mammography screening.

The incidence of uterine cancer began to significantly increase in 1999, according to national statistics, partly because of an increase in the incidence of cervical cancer,^16^ which had been previously decreasing. Incidences in some prefectures increased markedly in recent years (APC of 12.11 in Yamagata in 1998–2004, 15.43 in Niigata in 2000–2004, and 11.49 in Tottori in 1996–2004),^17^ and these regional rises increased the incidence in national estimates. It is necessary and important to continue to observe temporal trends in uterine cancer incidence in Fukui Prefecture. In this study, we also analyzed uterine cancer with respect to site, ie, cervical cancer or corpus cancer. There was a decline in the incidence of cervical cancer until 1988 (1984–88 APC, −13.80; significant), but in 1988–2004 there was an increasing trend (APC 1.21; not significant). Regarding uterine corpus cancer, the APC for incidence in 1984–2004 was 1.94 (not significant), indicating an increasing trend in Fukui Prefecture from 1984.

Regarding uterine cancer, the APC reduction in mortality was high; however, when we looked at cervical cancer and corpus cancer separately, the APC (−0.35, cervix, 1984–2004, not significant; −0.50, corpus, 1984–2004; not significant) in mortality moderately decreased, which was similar to the trend in national data for uterine cancer. Although a reduction in mortality mainly depends on a decrease in incidence, careful observation of future developments in Fukui is necessary. The incidence and mortality for uterine cancer of unspecified location were high: 9.6% (1.0% to 17.6%/year) and 40.6% (26.9% to 56.0%/year) on average, respectively. Because there were many such cases, we presume that there was greater dispersion in values, which explains the differences in the trends for uterine cancer and cervical/corpus cancer analyzed separately. In short, it is possible that there was statistical bias in the distribution of the population in the registry of uterine cancer.

In Fukui Prefecture, the annual percentage of eligible residents who undergo population-based mass screening for uterine cervical cancer was about 19% in 1989 and 14% in 2004. The ratio of localized uterine cancer remained stable: 45.3% in 1985 and 40.4% in 2002. The localized cancer/distant cancer ratio was 8.09 in 1994–1998, which was lower than that of Yamagata (11.79),^13^ and the 5-year relative survival rate for uterine cancer was 69.0% in 1985 and 72.2% in 1998. Because detection of early-stage cancer reached a plateau, it is possible that later cases have already presented with more advanced disease when detected, and that treatment outcomes thus do not improve. Cervical cancer affects younger women^16^; therefore, we should continue to monitor changes in the age at onset in patients, as well as discrepancies from the national data. We should also formulate policies that will enable us to detect uterine cancer at an early stage. Further development of uterine cancer examinations is now required.

The cancer detection rate for prostate cancer in 2003 was 1.48% by population-based mass screening using prostate-specific antigen (PSA) testing.^18^ According to the national estimates, the incidence of prostate cancer significantly increased, especially from 2000, although mortality remains unchanged (1999–2006). This may be due to increased screening through PSA testing, but in Fukui Prefecture, where both incidence and mortality have increased significantly, appropriate policies must be established, especially for reduction of mortality. The localized cancer/distant cancer ratio in 1994–1998 was 1.4 in Fukui, 1.1 in Yamagata, and 1.3 in Osaka,^13^ so Fukui has almost the same ratio of clinical stage distribution as the other 2 prefectures. The 5-year relative survival rate in 1985 was 35.6%, and improved to 62.4% in 1998. However, the 5-year relative survival rate for distant prostate cancer in 1994–1998 was 23.2% in Fukui, 35.4% in Yamagata, and 31.6% in Osaka.^13^ These findings explain why mortality in Fukui increased more quickly than in national data. These results suggest a need for early diagnosis by mass screening and a need for improved prostate cancer therapies in Fukui.

Cancer mortality has been declining in recent years. In particular, the reductions in mortality from stomach and uterine cancers have contributed to this trend. Using the method described in Cancer Statistics 2007,^19^ we calculated the contributions of individual cancer sites to the total decrease in overall cancer death rates (1990–2004). Stomach cancer was responsible for over 60% of the decrease in cancer death rates in both sexes, which confirms the impact of mass screening examinations and advances in diagnostic and therapeutic methods. For cancers of the colorectum (in men), liver, lung, breast, and prostate, there is a need for primary preventive measures—including smoking prevention—and improved secondary preventive measures, with an emphasis on early detection and treatment. We believe that the present findings will be useful for cancer control planning in local governments.
